# Isolation of *Debaryomyces hansenii* and selection of an optimal strain to improve the quality of low-grade beef rump (*middle gluteal*) during dry aging

**DOI:** 10.5713/ab.22.0475

**Published:** 2023-05-04

**Authors:** Yoonjeong Yoo, Hyemin Oh, Yohan Yoon

**Affiliations:** 1 Department of Food and Nutrition, Sookmyung Women’s University, Seoul 04310, Korea; 2 Risk Analysis Research Center, Sookmyung Women’s University, Seoul 04310, Korea

**Keywords:** *Debaryomyces hansenii*, Dry-aged Beef, Food Additive, Quality Improvement, Yeast

## Abstract

**Objective:**

The objective of this study was to evaluate the effect of *Debaryomyces hansenii* isolated from dry-aged beef on the tenderness and flavor attributes of low-grade beef during dry aging.

**Methods:**

Five *D. hansenii* strains were isolated from dry-aged beef samples. The rump of low-grade beef was inoculated with individual *D. hansenii* isolates and subjected to dry aging for 4 weeks at 5°C and 75% relative humidity. Microbial contamination levels, meat quality attributes, and flavor attributes in the dry-aged beef were measured.

**Results:**

Of the five isolates, the shear force of dry-aged beef inoculated with SMFM201812-3 and SMFM201905-5 was lower than that of control samples. Meanwhile, all five isolates increased the total free amino acid, glutamic acid, serine, glycine, alanine, and leucine contents in dry-aged beef. In particular, the total fatty acid, palmitic acid, and oleic acid contents in samples inoculated with *D. hansenii* SMFM201905-5 were higher than those in control samples.

**Conclusion:**

These results indicate that *D. hansenii* SMFM201905-5 might be used to improve the quality of beef during dry aging.

## INTRODUCTION

Fermented sausage and dry-aged meat are reported to contain yeast, which plays an important role in the aging process of meat products [1–3]. *Debaryomyces hansenii* (*D. hansenii*) is found in dry-aged meat [4,5]. The peptidases and proteases of yeast play a major role in the meat aging process. Consequently, yeast promotes enhanced protein degradation in dry-fermented sausages and increases the production of volatile compounds in dry-cured meat upon co-inoculation with other starter cultures [6–8].

During dry aging, the carcasses are exposed to air under refrigeration without packaging for 1 to 5 weeks, which increases the flavor of the meat [9,10]. The dry aging process imparts a strong taste and a unique flavor that cannot be sensed in meat subjected to other processing methods. Additionally, dry aging softens the meat [11].

During dry aging, the protein and fat components undergo chemical decomposition, which enhances the nutty flavor of the beef. Additionally, natural enzymes in beef degrade proteins and connective tissues in the muscles during dry aging [12]. Thus, dry-aged beef has a rich nutty and buttery flavor, excellent taste, and soft texture [13].

The use of microorganisms such as lactic acid bacteria, yeast, and mold during dry aging promotes the production of amino acids and fatty acids through the microbial degradation of proteins and fats [14–16]. Transamination and the subsequent decarboxylation of amino acids (branched, aromatic, and linear) result in the formation of respective aldehydes, alcohols, and acids, which impart aroma to meat [17]. In meat products, yeast is involved in the production of flavor compounds such as peptides, amino acids, fatty acids, and ester compounds, which improve the palatability of consumers [15,18]. Among yeast species, *D. hansenii* is known to have proteolytic and lipolytic activity, as well as the ability to ferment various sugars and degrade peroxides and amino acids [19]. *D. hansenii* is used as a starter in the production of dry-fermented sausage due to these characteristics [20].

In this study, *D. hansenii* was isolated from dry-aged beef. The effects of *D. hansenii* on the tenderness and flavor profiles of low-grade beef were examined.

## MATERIALS AND METHODS

### Isolation of *D. hansenii* from dry-aged beef

Thirty-one pieces of dry-aged beef manufactured by 11 different companies were purchased from an online shopping mall in South Korea. The dry-aged beef samples (25 g) were placed in sterile bags and homogenized with 50 mL of 0.1% buffered peptone water (BPW; Becton Dickinson, Franklin Lakes, NJ, USA) for 60 s using a pummeller (BagMixer 400 W; Interscience, St. Nom, France). To isolate yeast colonies, the homogenates were diluted 10-fold with BPW. Aliquots (0.1 mL) of the diluted homogenate were spread-plated onto potato dextrose agar (PDA; Becton Dickinson, USA) supplemented with 10% tartaric acid (Samchun, Seoul, Korea). The samples were incubated at 20°C for 48 h. White and round colonies (typical morphological characteristics of *D. hansenii*) were subjected to 26S rRNA sequencing analysis. The D1 and D2 domains of the 26S rRNA were amplified with the following primers: NL1 (5′-GCATATCAATAAGCGG AGGAAAAG-3′) and NL4 (5′-GGTCCGTGTTTCAAGA CGG-3′). Sequencing was performed at COSMOGENETECH (Seoul, Korea). The 26S rRNA sequence of the strains was compared with that obtained from the National Center for Biotechnology Information (NCBI). The identified strains were cultured in 10 mL potato dextrose broth (PDB; Becton Dickinson, USA) supplemented with 10% tartaric acid and stored at −70°C as glycerol (20%) stocks.

### Safety analysis of *D. hansenii* inoculation

*Hemolysis*: Hemolytic properties were examined, following the guidelines of the American Society for Microbiology [21]. The cultured *D. hansenii* isolates (SMFM201812-1, SMFM201812-3, SMFM201905-4, SMFM201905-5, and SMFM201905-15) were streaked onto Columbia agar plates containing 5% sheep blood (bioMerieux, Marcy l’Etoile, France) and incubated at 20°C for 48 h to evaluate their hemolytic activities. Strains without a clear zone around the colonies were considered non-hemolytic. Meanwhile, strains with partially clear zones around the colonies with green color were considered to exhibit *α*-hemolysis, whereas those with clear transparent zones around the colonies were considered to exhibit *β*-hemolysis.

*Cytotoxicity*: The cytotoxicity was measured by modifiying the method of Sadeghi-Aliabadi et al [22]. HT-29 cells were cultured in Dulbecco’s modified Eagle’s medium (DMEM; Welgene, Gyeongsan, Korea) supplemented with 1% penicillin-streptomycin (Welgene, Korea) and 10% fetal bovine serum (Gibco, Grand Island, NY, USA) at 37°C and 5% CO_2_. The cells were subcultured when more than 80% were attached to the T-75 flask. For trypsinization, cells were incubated with 3 mL trypsin (Gendepot, Barker, TX, USA) for 3 min. The trypsinized cells were centrifuged at 217 *g* and 25°C for 5 min. The supernatant was discarded, and the pellet was resuspended in 1 mL of DMEM. The cell suspension (1 mL) was transferred to a new T-75 flask and cultured in 19 mL of fresh DMEM. When more than 80% of cells were attached to the 75-T flask, the subcultured cells were treated with 3 mL of trypsin for 3 min. The cell suspension was centrifuged at 217 *g* and 25°C for 5 min. Next, the cell pellet was washed with the medium and resuspended in DMEM. The cells were then seeded in a 96-microtiter plate at a density of 5×10^4^ cells/mL and incubated at 35°C with 5% CO_2_ for 24 h. In individual wells of the microtiter plate, 20 μL of the *D. hansenii* isolates (5 log CFU/mL) were incubated with 180 μL of DMEM without antibiotics at 37°C and 5% CO_2_ for 24 h. The culture supernatant was carefully removed from each well using a micropipette. The samples were then incubated with 5 mg/mL of 3-(4,5-dimethyl-2-thiazolyl)-2,5-diphenyl-2H-tetrazolium bromide (MTT; Sigma-Aldrich, St Louis, MO, USA) reagent at 37°C and 5% CO_2_ for 4 h to allow the metabolism of MTT by HT-29 cells. The supernatant was removed using a micropipette, and the samples were solubilized in dimethyl sulfoxide (Samchun, Korea). The absorbance of the mixture at 540 nm (OD_540_) was measured using a microplate spectrophotometer (BioTek, Winooski, VT, USA). Cytotoxicity was evaluated by measuring cell viability, and cell viability was calculated using the following equation:


$$\text{Cell viability }(\%)=({\text{OD}}_{540}\;\text{of the sample}/{\text{OD}}_{540}\;\text{of the control})\times 100$$

*Analysis of D. hansenii-inoculated beef quality during dry aging production of dry-aged beef*: *D. hansenii* isolates (SMFM 201812-1, SMFM201812-3, SMFM201905-4, SMFM201905-5, and SMFM201905-15) (100 μL) stored at −70°C were inoculated into 10 mL PDB and cultured at 20°C for 72 h. For subculturing, 0.1 mL aliquots of the cultures were inoculated into 10 mL fresh PDB and cultured at 20°C for 72 h. The subculture was transferred to a 15-mL conical tube and centrifuged at 1,912 *g* and 4°C for 15 min. The cells were washed twice with phosphate-buffered saline (PBS, pH 7.4; 8.0 g NaCl, 1.5 g NaHPO_4_, 0.2 g KH_2_PO_4_, 0.2 g KCl in 1 L distilled water). The cell density of the suspension was adjusted to 6 log CFU/mL before inoculation. The cattle (22 month old Holstein steer) was slaughtered in Anseong, Korea on August 6, 2019. The grade of the beef was grade 2 which is the fourth grade out of five grades (1++, 1+, 1, 2, and 3) in South Korea. The rump beef within 1 week after slaughtering was subjected to UV irradiation for 10 min in a bio cabinet. Next, rump beef (600 g) was evenly sprayed with 3 mL of five *D. hansenii* isolates using a sprayer. The control samples were sprayed with PBS. The inoculated beef sample was incubated at room temperature for 15 min to allow *D. hansenii* attachment and subjected to dry aging at 5°C and 75% relative humidity in a thermo-hygrostat (KCL-2000; EYELA, Tokyo, Japan) for 4 weeks.

*Microbial analysis*: Microbial analysis of dry-aged beef crusts was performed after 4 weeks of dry aging. After trimming the crust, 10 g of dry-aged meat crust and 20 mL of 0.1% BPW were transferred to a filter bag (3M, St. Paul, MN, USA). The samples were homogenized for 60 s using a pummeller (BagMixer 400 W; Interscience, France). The homogenate was serially diluted with 0.1% BPW. Each dilution (0.1 mL) was plated onto tryptic soy agar (TSA; Becton Dickinson, USA) or PDA supplemented with 10% tartaric acid. The samples plated onto TSA plates were incubated at 37°C for 24 h to determine the total aerobic bacterial counts. To enumerate yeast, each dilution of the sample was plated onto PDA supplemented with 10% tartaric acid and cultured at 20°C for 48 h.

*pH*: Before measurement, the pH meter was calibrated using pH 4.01, pH 7.00, and pH 10.01 buffered solution (Thermo Fisher Scientific Inc., San Jose, CA, USA), and the pH was measured by modifying the method of Calicioglu et al [23]. The dry-aged beef sample (10 g) along with 0.1% BPW (20 mL) was transferred to sterile sample bags and homogenized for 60 s using a pummeller. The pH value of the homogenate was measured using a pH meter (Thermo Fisher Scientific Inc., USA).

*Anlaysis of thiobarbituric acid reactive substance*: Lipid rancidity was measured following the methods described by Park et al [24] and Hwang et al [25]. The samples (1 g) along with 3 mL distilled water were placed in sterile sample bags and homogenized for 60 s using a pumeller. Next, 1 mL aliquots of the homogenate without solid contents were incubated with 2 mL of 20 mM 2-thiobarbituric acid/20% trichloroacetic acid at 100°C for 15 min in a water bath. The reaction was stopped by cooling the reactant in flowing water. After filtering the supernatants with No. 1 filter paper (Advantec, Tokyo, Japan), the absorbance of the filtrate at 531 nm was measured using an Epoch microplate spectrophotometer (BioTek, USA).

*Shear force*: The beef sample (100 g) was heated upto 70°C the core temperature followed by cooling under running water. The sample was cut into pieces (1 cm×1 cm ×4 cm) perpendicular to the direction of the muscle fiber. The shear force was measured using a texture analyzer (TA-XT2; Stable Micro Systems Ltd., Haslemere, UK) equipped with a Warner-Bratzler shear blade. The test conditions were as follows: pre-test speed 2.0 mm/s, test speed 2.0 mm/s, post-test speed 8.0 mm/s, and distance 30.0 mm. Measurements were performed until the sample was completely cut perpendicular to the direction of the muscle fiber.

*Free amino acid analysis*: The sample (3 g) was incubated with 15 mL of 0.01 N HCl and homogenized using a pummeller for 1 min. The homogenate was filtered through a No. 1 filter paper (Advantec, Japan). The filtrate (600 μL) was incubated with 20 μL of internal standard solution (1 mg/mL L-citrulline; Sigma-Aldrich, USA) and 1,380 μL of acetonitrile. The mixture was left undisturbed for 30 min and centrifuged at 10,000 *g* for 15 min. The supernatant was filtered through a 0.2-μm syringe filter. Free amino acid content was analyzed using a Dionex Ultimate 3000 HPLC system (Thermo Fisher Scientific Inc., USA) at the National Instrumentation Center for Environmental Management (Seoul, Korea) as follows: column, Inno C18 column (4.6 mm×150 mm, 5 μm); column temperature, 40°C; injection volume, 0.5 μL; mobile phase A, 40 mM sodium phosphate (pH 7); mobile phase B, distilled water/acetonitrile/methanol = 10%/45%/45% (v/v).

*Fatty acid analysis*: The sample (1 g) was mixed with 2 mL of pyrogallol, 2 mL of an internal standard solution (triundecanoin), and 10 mL of 8.3 M HCl, and the mixture was sealed with parafilm. To promote decomposition, the samples were incubated at 70°C to 80°C for 40 min in a drying oven. The decomposed product was incubated with 25 mL of ethyl ether for 5 min with shaking, followed by incubation with 25 mL of petroleum ether for 5 min with shaking. The sample was evaporated at 35°C to 40°C using a nitrogen concentrator, vortex-mixed with 2 mL of chloroform and 3 mL of ethyl ether, and concentrated at 40°C using a nitrogen microconcentrator. The concentrate was incubated with 2 mL of 7% BF_3_-methanol and 1 mL of toluene at 100°C for 45 min, followed by incubation at room temperature. Next, the sample was incubated with 5 mL distilled water, 1 mL isooctane, and 1 g Na_2_SO_4_; vortexed; and centrifuged at 448 *g* for 5 min. The supernatant was used as a test solution. Gas chromatography was performed using Agilent 7890A (Agilent, Palo Alto, CA, USA) with an SP-2560 (100 m×0.25 mm×0.2 μm) column. The chromatography conditions were as follows: sample inlet temperature, 225°C; detector temperature, 285°C; and mobile phase flow rate, 0.75 mL/min. The results of the test samples were compared with those of the standard solution (a fatty acid standard: isooctane). The analysis was performed at the Food Analysis Research Center (Suwon, Korea).

### Statistical analysis

The experiment was repeated three times, and every replication used beef from different carcasses. Dry-aged beef samples treated with six treatments were analyzed on week 0 and week 4. Data were analyzed using the general linear model procedure of SAS (version 9.3, SAS Institute Inc., Cary, NC, USA). Significant differences in least squares means were determined by a pairwise *t*-test at *α* = 0.05.

## RESULTS AND DISCUSSION

### Genetic characteristics of *D. hansenii* isolated from dry-aged beef

The isolates from dry-aged beef were identified using 26S rRNA sequencing. The following microorganisms were identified in the samples: yeasts such as *Debaryomyces hansenii*, *Yarrowia lipolytica*, *Serratia* spp., *Candida zeylanoides*, *Debaryomyces maramus*, *Pantoea agglomerans*, and *Candida sake*; molds such as *Pilaira anomala*, *Mucor circinelloides*, *Mucor velutinosus*, and *Trichosporon pullulans*; lactic acid bacteria such as *Leuconostoc gelidum*, *Lactobacillus sakei*, *Carnobacterium gallinarum*, and *Carnobacterium divergens*. Yeasts whose pathogenecity has not been evaluated were excluded. Accordingly, *D. hansenii* was selected for further experiments evaluating the effects of *D. hansenii* on beef quality during dry aging. Five *D. hansenii* isolates (SMFM 201812-1, SMFM201812-3, SMFM201905-4, SMFM201905-5, and SMFM201905-15) were identified in this study.

### Safety of *D. hansenii* isolates

*Hemolytic property*: Strains without a clear zone around their colonies were considered non-hemolytic. However, strains with a green-colored colony and a transparent clear zone were considered to exhibit *α*-hemolytic and *β*-hemolytic properties, respectively. None of the *D. hansenii* isolates formed clear zones on Columbia agar with 5% sheep blood or green-colored colonies. This indicates that the isolates did not exhibit hemolytic activity.

**Figure 1:**
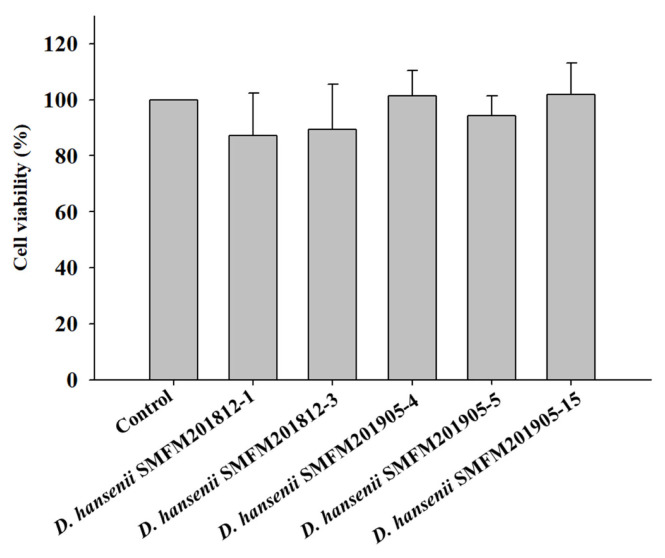
Effect of *Debaryomyces hansenii* isolates on the viability of HT-29 cells using MTT assay. Data are expressed as mean and standard deviation from three replications. MTT, 3-(4,5-dimethyl-2-thiazolyl)-2,5-diphenyl-2H-tetrazolium bromide.

*Cytotoxicity*: Cytotoxicity of the isolates against an intestinal epithelial cell line (HT-29 cells) was evaluated and isolates exerting low cytotoxicity were selected. In the MTT assay, yellow-colored MTT penetrates the viable cells where it is converted into purple-colored formazan by mitochondrial reductase, thereby confirming viable cells [26]. The percentage viability of HT-29 cells infected with *D. hansenii* isolates was in the range of 87.1% to 101.8% (Figure 1). However, the percentage viability was not significantly different between cells individually infected with five *D. hansenii* isolates and control cells. This suggested that *D. hansenii* isolates did not exert cytotoxic effects against HT-29 cells.

### Effects of *D. hansenii* isolates on beef quality during dry aging

*Bacterial counts in dry-aged beef crust*: The initial counts of total aerobic bacteria and yeast were 4.1 log CFU/g and 2.6 log CFU/g, respectively. The total aerobic bacterial counts in the crust varied from 3.3 log CFU/g to 6.8 log CFU/g after 4 weeks of aging depending on the isolates. After 4 weeks of dry aging, the yeast cell counts in dry-aged beef samples inoculated with the *D. hansenii* isolates were higher than those in the control dry-aged beef samples (Table 1). These *D. hansenii* isolates in beef may improve flavor of dry-aged meat by the protease and lipase of microorganisms [5], because the enzymes cleave peptidic bonds in muscle proteins, causing muscle structure degradation and increasing meat tenderness as the aging process progresses [27].

*pH*: One of the major factors affecting beef quality is the pH as it affects quality parameters, such as the water-holding capacity and tenderness [28]. The pH of raw beef, which is between 5.6 and 5.8, gradually increases with the increase in the storage period [29]. After 4 weeks of dry aging, the pH values were in the range of 5.70 to 5.82. However, the pH values were not significantly different among the treatment groups. Increased pH by spoilage bacteria may catalyze proteins to amino acids and volatile organic compounds [30]. The pH values were higher than 6.2 to 6.3, which indicates early spoilage in meat [29]. These findings suggest that dry-aged beef inoculated with *D. hansenii* did not undergo spoilage.

**Figure 2:**
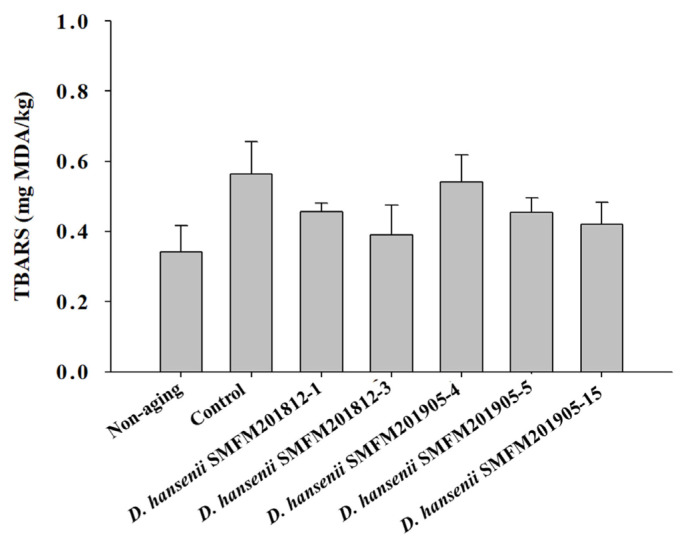
Thiobarbituric acid reactive substance (TBARS) values of dry-aged beef inoculated with different *Debaryomyces hansenii* isolates for 4 weeks. Data are reported as mg malondialdehyde (MDA) per kg as mean and standard deviation from three replications.

*Thiobarbituric acid reactive substance*: Rancidity is determined based on the thiobarbituric acid reactive substance (TBARS) values. The TBARS value of raw beef was 0.34 mg of malonaldehyde (MDA)/kg, while that of dry-aged beef was in the range of 0.39 to 0.56 mg of MDA/kg (Figure 2). In this study, the control group had the highest TBARS value after dry aging, but there was no significant difference between experimental groups of dry-aged beef and beef not treated to dry-aging (Figure 2). This indicated that *D. hansenii* isolates did not enhance the rancidity of dry-aged beef. Lowered lipid oxidation by yeast was observed in several studies [18, 31,32]. Flores et al [32] suggested that *D. hansenii* could lower the lipid oxidation and the formation of ethyl esters in the fermented meat.

**Figure 3:**
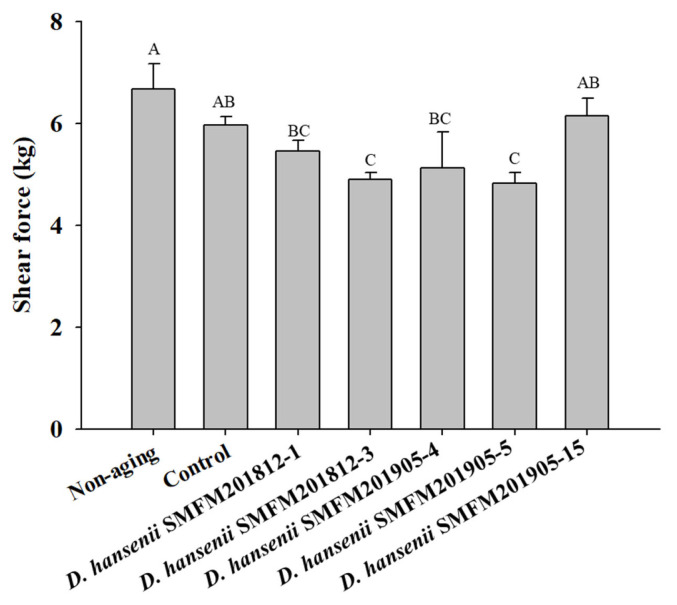
Shear force of dry-aged beef inoculated with *Debaryomyces hansenii* isolates for 4 weeks. Data are expressed as mean and standard deviation from three replications. ^A–C^ Different letters indicate significant differences among means at p<0.05.

*Shear force*: After 4 weeks of dry aging, the shear force of the control group was 5.97 kg, which was significantly higher (p<0.05) than that of the dry-aged beef samples inoculated with strains SMFM201812-3, SMFM201905-4, and SMFM 201905-5 (Figure 3). Among the several sensory and physical factors that positively influence beef consumption, tenderness is the major factor affecting the sensory quality of meat. Shear force measurement is the most representative method for determining tenderness [33,34]. In this study, *D. hansenii* strains SMFM201812-3, SMFM201905-4, and SMFM201905-5 decreased the shear force of beef samples. *D. hansenii* is known to have lipolytic and proteolytic activities that can affect shear force [8]. Dashdorj et al [12] and Lee et al [35] reported that proteolytic and collagenolytic enzymes could reduce the shear force of the meat after dry-aging. As the enzymes penetrate the meat, muscle and connective tissue could be broken down. However, the level of lipolytic and proteolytic activity was dependent on the yeast strains [36]. As for the treated strains, the 3 strains (SMFM201812-3, SMFM201905-4, and SMFM 201905-5) are considered to have higher lipolytic and proteolytic activities and thus, lower shear force than other strains.

*Free amino acid composition*: Based on the results of pH, TBARS, and shear force analyses, SMFM201812-3 and SMFM 201905-5 were selected as the most effective isolates for improving beef quality during dry aging. The effects of SMFM 201812-3 and SMFM201905-5 on the free amino acid and fatty acid contents in the dry-aged beef samples were examined. The total free amino acid, glutamic acid, serine, glycine, alanine, and leucine contents increased in the groups inoculated with SMFM201812-3 and SMFM201905-5 strains and were higher than those in the control group (Table 2). The contents of free amino acids increased during beef aging due to the proteolytic enzyme-mediated degradation of myofibril proteins in beef [37]. Free amino acids, which are flavor component precursors, generate various flavor components through the Maillard reaction and Strecker degradation, as well as reducing sugar components, when meat is heated [38]. Glutamic acid and aspartic acid, which are reported to produce beefy and/or umami flavors in beef [39], are considered one of the primary contributors to the enhanced flavor of dry-aged beef products. Alanine and glycine have a sweet taste, whereas serine and proline have sweet and bitter tastes. The leucine content in dry-aged beef is higher than that in wet-aged beef. Leucine and isoleucine, in particular, can interact with dicarbonyl compounds produced by the Maillard reaction to produce meat odorants such as 2- and 3-methylbutanal [40,41]. Hydrolysis of meat proteins results in the production of small peptides and polypeptides that can be further degraded into free amino acids. *Debaryomyces* exhibited aminopeptidase activity, which can affect the flavor of aged meat by degrading meat proteins [7,42]. The enzymatic activity of the *D. hansenii* isolates was confirmed using the API ZYM kit (bioMerieux, France). SMFM 201812-3 and SMFM201905-5 exhibited leucine arylamidase activities, which affected increase in leucine concentrations in dry-aged beef inoculated with SMFM201812-3 and SMFM 201905-5 (Supplementary Table S1). Therefore, during dry aging, the production of free amino acids may be accelerated by the enzymes of SMFM201812-3 and SMFM201905-5.

*Fatty acid composition*: Total fatty acid, palmitic acid, palmitoleic acid, oleic acid, and linoleic acid contents in the groups inoculated with SMFM201812-3 and SMFM201905-5 were higher than those in the control group after 4 weeks of dry aging (Table 3).

In particular, total fatty acid, palmitic acid, and oleic acid contents in the group inoculated with SMFM201905-5 were significantly higher (p<0.05) than those in the control group (Table 3). Several studies have reported increased total fatty acid contents during lipolysis upon *D. hansenii* inoculation for dry sausage production [8,40]. The results of our study are consistent with those of the previous studies [8,43]. Even more, *D. hansenii* is well known yeast that can produce lipid it self [41,42]. The enzyme ATP citrate lyase is the key factor in the biosynthesis of fatty acids [44,45]. This enzyme generates acetyl-CoA from citrate, the key substrate for fatty acid biosynthesis, in the cytoplasm [44,45]. Thus, this characteristic of *D. hansenii* may contribute the increased total fatty acid content in our study.

Moreover, SMFM201812-3 and SMFM201905-5 exhibited esterase and esterase lipase activities (Supplementary Table S1). Esterase and esterase lipase, which are involved in the esterification reaction of alcohol and acid, can enhance the flavor of aged meat through ester generation [46]. Fatty acids in meat are known to enhance the flavor and enrich the taste as they are associated with juiciness. Palmitic acid content is positively correlated with consumer preference, while oleic acid content is positively correlated with taste, juiciness, and tenderness [27,47]. This indicates that *D. hansenii* SMFM 201905-5 increased the contents of fatty acids associated with meat flavor, especially palmitic acid and oleic acid contents, during dry aging.

## CONCLUSION

Among the *D. hansenii* isolates examined in this study, SMFM 201905-5 improved the quality of dry-aged beef by decreasing the shear force and increasing the contents of flavor-related free amino acids and fatty acids.

## Figures and Tables

**Table 1:** Cell counts of total aerobic bacteria and yeast in crust of dry-aged beef inoculated with *Debaryomyces hansenii* isolates for 4 weeks

Treatment	Total aerobic bacteria (log CFU/g)	Yeast (log CFU/g)
Control	6.7±1.1^A^	6.5±0.5^B^
*D. hansenii* SMFM201812-1	5.8±3.2^AB^	7.0±0.7^AB^
*D. hansenii* SMFM201812-3	6.8±0.5^A^	7.3±0.2^AB^
*D. hansenii* SMFM201905-4	6.6±1.3^A^	7.7±0.5^A^
*D. hansenii* SMFM201905-5	3.3±0.5^B^	6.7±0.7^AB^
*D. hansenii* SMFM201905-15	5.7±2.0^AB^	7.3±0.4^AB^

Data are presented as mean±standard deviation.

A,B Different letters in a column indicate significant differences among means at p<0.05.

**Table 2:** Free amino acid concentration (mean±standard deviation) of dry-aged beef inoculated with *Debaryomyces hansenii* after 4 weeks of dry-aging

Free amino acid (mg/100 g)	Non aging1)	Control1)	SMFM201812-31)	SMFM201905-51)
Aspartic acid	0.37±0.06^B^	1.39±0.81^AB^	1.71±0.89^A^	0.86±0.16^AB^
Glutamic acid	6.64±1.56^B^	11.75±1.34^B^	35.80±11.46^A^	34.27±7.99^A^
Asparagine	2.35±0.42^B^	4.91±0.57^B^	14.31±2.16^A^	13.04±2.70^A^
Serine	3.25±0.26^B^	7.46±0.72^B^	24.17±2.55^A^	22.41±4.87^A^
Glutamine	76.01±4.09	82.65±24.78	95.40±37.08	67.42±11.00
Histidine	1.76±0.35^B^	3.25±0.39^B^	9.05±2.00^A^	7.40±1.80^A^
Glycine	3.48±0.88^B^	6.09±1.22^B^	18.87±2.95^A^	16.37±2.98^A^
Threonine	4.12±0.43^B^	8.10±0.42^B^	19.74±1.91^A^	17.72±4.38^A^
Arginine	5.36±0.10^B^	6.13±0.62^B^	19.24±3.26^A^	17.85±4.40^A^
Alanine	20.59±7.33^B^	26.42±5.24^B^	91.05±12.62^A^	72.62±12.52^A^
Tyrosine	2.71±0.08^B^	5.92±0.27^B^	16.84±0.72^A^	15.62±3.88^A^
Valine	3.75±0.74^B^	7.49±0.28^B^	26.74±0.49^A^	24.46±5.82^A^
Methionine	1.55±0.23^B^	3.74±0.41^B^	13.16±0.26^A^	12.70±2.80^A^
Tryptophane	2.26±0.59^C^	4.33±0.58^B^	9.33±0.71^A^	8.14±1.79^A^
Phenylalanine	3.61±0.61^B^	5.19±0.38^B^	17.92±0.78^A^	16.91±3.86^A^
Isoleucine	2.52±0.29^B^	4.77±0.44^B^	18.51±0.35^A^	17.59±4.06^A^
Leucine	5.04±0.81^B^	9.26±0.82^B^	33.79±1.59^A^	32.30±7.71^A^
Lysine	3.88±0.79^B^	6.03±1.25^B^	24.45±3.59^A^	21.51±5.71^A^
Proline	1.59±0.67^B^	2.35±0.95^B^	6.93±1.76^A^	8.55±2.00^A^
Total free amino acid	150.86±9.72^B^	207.25±37.66^B^	497.00±77.57^A^	427.75±88.37^A^

1) Non aging, non-inoculated group before dry-aging; Control, non-inoculated group; SMFM201812-3, *D. hansenii* SMFM201812-3 inoculated group; SMFM201905-5, *D. hansenii* SMFM201905-5 inoculated group.

A–C Different letters within a row indicate significant differences among means at p<0.05.

**Table 3:** Fatty acid concentration (mean±standard deviation) of dry-aged beef inoculated with *Debaryomyces hansenii* after 4 weeks of dry-aging

Fatty acid (m/100 g)	Control1)	SMFM201812-31)	SMFM201905-51)
Butyric acid (C4:0)	0.000±0.000	0.000±0.000	0.000±0.000
Caproic acid (C6:0)	0.000±0.000	0.000±0.000	0.000±0.001
Caprylic acid (C8:0)	0.000±0.000	0.000±0.000	0.000±0.001
Capric acid (C10:0)	0.003±0.000^B^	0.004±0.002^AB^	0.008±0.003^A^
Undecanoic acid (C11:0)	0.000±0.000	0.000±0.000	0.000±0.000
Lauric acid (C12:0)	0.005±0.001	0.006±0.004	0.011±0.005
Tridecanoic acid (C13:0)	0.000±0.000	0.000±0.000	0.001±0.002
Myristic acid (C14:0)	0.134±0.041^B^	0.216±0.181^AB^	0.458±0.209^A^
Myristoleic acid (C14:1)	0.064±0.009	0.076±0.071	0.163±0.081
Pentadecanoic acid (C15:0)	0.016±0.001	0.016±0.012	0.033±0.015
Pentadecenoic acid (C15:1)	0.000±0.000	0.000±0.000	0.000±0.000
Palmitic acid (C16:0)	1.139±0.096^B^	1.874±1.318^AB^	3.786±1.555^A^
Palmitoleic acid (C16:1)	0.234±0.017	0.275±0.225	0.573±0.26
Margaric acid (C17:0)	0.043±0.002	0.053±0.038	0.108±0.051
Heptaecenoic acid (C17:1)	0.000±0.000	0.000±0.000	0.000±0.000
Stearic acid (C18:0)	0.338±0.383^B^	0.794±0.452^AB^	1.523±0.638^A^
Oleic acid (C18:1,trans)	0.078±0.025	0.110±0.050	0.202±0.098
Oleic acid (C18:1,cis)	1.861±0.075^B^	3.041±2.093^AB^	6.152±2.797^A^
Linoleic acid (C18:2,trans)	0.015±0.003	0.020±0.011	0.047±0.034
Linoleic acid (C18:2,cis)	0.208±0.050^B^	0.294±0.051^AB^	0.361±0.093^A^
Arachidic acid (C20:0)	0.002±0.001^B^	0.006±0.003^AB^	0.010±0.004^A^
γ-linolenic acid (C18:3n-6)	0.000±0.000	0.000±0.000	0.000±0.000
Linolenic acid (C18:3n-3)	0.013±0.007	0.008±0.003	0.014±0.005
Gadoleic acid (C20:1)	0.007±0.001^B^	0.015±0.008^AB^	0.030±0.013^A^
Heneicosanoic acid (C21:0)	0.008±0.000^B^	0.016±0.012^B^	0.037±0.022^A^
Eicosadienoic acid (C20:2)	0.003±0.001^B^	0.004±0.002^AB^	0.008±0.004^A^
Behenic acid (C22:0)	0.000±0.000^B^	0.002±0.000^A^	0.002±0.001^A^
Dihomo-γ-linolenic acid (C20:3n-6)	0.019±0.006^B^	0.037±0.003^A^	0.037±0.004^A^
Erucic acid (C22:1n-9)	0.000±0.000	0.000±0.000	0.000±0.001
Eicosatrienoic acid (C20:3n-3)	0.000±0.000	0.000±0.000	0.000±0.000
Arachidonic acid (C20:4n-6)	0.060±0.174^B^	0.093±0.009^A^	0.080±0.004^AB^
Tricosanoic acid (C23:0)	0.000±0.000^B^	0.001±0.000^A^	0.001±0.001^A^
Brassic acid (C22:2)	0.000±0.000	0.000±0.000	0.000±0.000
Lignoceric acid (C24:0)	0.000±0.000^B^	0.002±0.000^A^	0.002±0.001^A^
EPA (C20:5n-3)	0.000±0.000^B^	0.002±0.000^A^	0.001±0.001^A^
Nervonic acid (C24:1)	0.003±0.001	0.003±0.001	0.005±0.002
DHA (C22:6n-3)	0.000±0.000	0.000±0.000	0.000±0.000
Total fatty acid	4.306±0.225^B^	6.970±4.524^AB^	13.655±5.894 ^A^

EPA, eicosapentaenoic acid; DHA, docosahexaenoic acid.

1) Control, non-inoculated group; SMFM201812-3, *D. hansenii* SMFM201812-3 inoculated group; SMFM201905-5, *D. hansenii* SMFM201905-5 inoculated group.

A,B Different letters within a row indicate significant differences among means at p<0.05.

## SUPPLEMENTARY MATERIAL

**Supplementary Table S1**. Enzyme profile of Debaryomyces hansenii isolates
