# Supplementary material for: Isolation of Debaryomyces hansenii and selection of an optimal strain to improve the quality of low-grade beef rump (middle gluteal) during dry aging
Source: Anim Biosci. 2023 May 4;36(9):1426–34. doi: 10.5713/ab.22.0475 (PMC10472159; doi:10.5713/ab.22.0475)
Supplement: Supplementary file 1 [file ab-22-0475-Supplementary-Table-1.pdf]

Supplementary materials

Table S1. Enzyme profile of *Debaryomyces hansenii* isolates

Enzyme	SMFM 201812-1	SMFM 201812-3	SMFM 201905-4	SMFM 201905-5	SMFM 201905-15
Alkaline phosphatase	0	0	0	0	0
Esterase	3	3	3	3	3
Esterase lipase	2	3	2	2	3
Lipase	0	0	0	0	0
Leucine arylamidase	4	4	4	4	5
Valine arylamidase	0	0	0	0	1
Cystine arylamidase	0	0	0	0	0
Trypsin	0	0	0	0	0
α-chymotrypsin	0	0	0	0	0
Acid phosphatase	0	0	0	0	5
Naphthol-AS-BI-phosphohydrolase	4	4	4	4	4
α-galactosidase	0	0	0	0	0
β-galactosidase	0	0	0	0	0
α-glucosidase	0	0	3	3	0
β-glucosidase	0	0	1	0	0
N-acetyl-β-glucosaminidase	0	0	0	0	0
α-mannosidase	0	0	0	0	0
α-fucosidase	0	0	0	0	0
